# Proteasome Inhibitors Interrupt the Activation of Non-Canonical NF-κB Signaling Pathway and Induce Cell Apoptosis in Cytarabine-Resistant HL60 Cells

**DOI:** 10.3390/ijms23010361

**Published:** 2021-12-29

**Authors:** Shuo-Yu Wang, Yin-Hwa Shih, Tzong-Ming Shieh, Yu-Hsin Tseng

**Affiliations:** 1 Department of Pediatrics, Kaohsiung Medical University Hospital, Kaohsiung 80756, Taiwan; wangcida@hotmail.com; 2 Department of Pediatrics, School of Medicine, College of Medicine, Kaohsiung Medical University, Kaohsiung 80708, Taiwan; 3 Department of Healthcare Administration, Asia University, Taichung 41354, Taiwan; evashih@asia.edu.tw; 4 School of Dentistry, China Medical University, Taichung 40402, Taiwan; tmshieh@mail.cmu.edu.tw; 5 Department of Dental Hygiene, China Medical University, Taichung 40402, Taiwan

**Keywords:** proteasome inhibitors, cytarabine-resistant HL60, NF-κB, leukemia

## Abstract

Over half of older patients with acute myeloid leukemia (AML) do not respond to cytotoxic chemotherapy, and most responders relapse because of drug resistance. Cytarabine is the main drug used for the treatment of AML. Intensive treatment with high-dose cytarabine can increase the overall survival rate and reduce the relapse rate, but it also increases the likelihood of drug-related side effects. To optimize cytarabine treatment, understanding the mechanism underlying cytarabine resistance in leukemia is necessary. In this study, the gene expression profiles of parental HL60 cells and cytarabine-resistant HL60 (R-HL60) cells were compared through gene expression arrays. Then, the differential gene expression between parental HL60 and R-HL60 cells was measured using KEGG software. The expression of numerous genes associated with the nuclear factor κB (NF-κB) signaling pathway changed during the development of cytarabine resistance. Proteasome inhibitors inhibited the activity of non-canonical NF-κB signaling pathway and induced the apoptosis of R-HL60 cells. The study results support the application and possible mechanism of proteasome inhibitors in patients with relapsed or refractory leukemia.

## 1. Introduction

Acute myeloid leukemia (AML) is one of the most common acute leukemias in adults [1,2,3]. Cytarabine, a pyrimidine nucleoside analog, is the main drug used for the treatment of AML [4]. Typical treatment processes for AML comprise two main phases, namely intensive induction therapy and consolidation therapy. The standard induction therapy, consisting of conventional doses of cytarabine for seven days and anthracycline for three days, achieves an AML remission rate of over 70%. However, only 40% of patients receiving this therapy become long-term survivors; most relapse because of the development of drug resistance [3,5,6]. Intensive treatment with high-dose cytarabine can increase the overall survival rate and reduce the relapse rate of AML, but it also increases the likelihood of drug-related side effects [7]. Elderly patients with AML often respond poorly to standard induction therapy because of the high frequency of adverse genomic features and increased resistance to treatments [8]. Therefore, elderly patients often receive less-intensive regimens, including those involving hypomethylating agents (azacitidine or decitabine) and low-dose cytarabine. Moreover, studies have indicated that overall survival was longer, and the incidence of remission was higher among patients who received azacitidine plus venetoclax than among those who received azacitidine alone [9]. However, for patients who reach the complete remission phase, consolidation therapy remains a necessary step to prevent recurrence. High dose cytarabine is the main chemotherapy option for consolidation therapy. In addition, for selected “highly fit” older adults, high to intermediate dose cytarabine remains the backbone of any consolidation approach [10]. Although numerous trials have been conducted to investigate different induction and consolidation therapy regimens, no established consensus or guidelines regarding the optimal treatment of patients with relapsed or refractory leukemia are available. Acquired resistance to anticancer agents is the main obstacle to successful cancer treatment and overcoming resistance to cytarabine could enable new strategies for the treatment of AML. To optimize the treatment of cytarabine, understanding the mechanism of resistance to cytarabine in leukemia cells is necessary. The principal mechanism underlying resistance to cytarabine appears to be related to insufficient cellular uptake and retention of cytarabine, overexpression of the enzymes inactivating cytarabine, increased cellular deoxycytidine triphosphate (dCTP) pools, and increased DNA repair [11]. For cytarabine to function as an antitumor agent, it must be transported into leukemia cells through membrane transporters such as the human equilibrative nucleoside transporter 1 (hENT1) [12]. Inside the cells, cytarabine is phosphorylated into cytarabine monophosphate by the rate-limiting enzyme deoxycytidine kinase (dCK) and then phosphorylated into cytarabine diphosphate by deoxycytidine monophosphate kinase and finally modified into cytarabine triphosphate by nucleoside diphosphate kinase [12]. Cytarabine triphosphate is then incorporated into DNA strands during the S phase of the cell cycle, thereby inhibiting DNA synthesis [5]. Overall, hENT1 and dCK play pivotal roles in cytarabine resistance in leukemic cells, and reductions in their expression have been reported to be involved in cytarabine resistance [12,13,14,15].

However, some patients with AML exhibit high hENT1 or dCK levels after relapse [13]. Additionally, one study demonstrated that two cytarabine-resistant cell lines (CEM/4 X AraC and CEM/20 X AraC) derived from the same human leukemia cells have distinct cytarabine resistance mechanisms. In CEM/4 X AraC cells, the *SLC29A1* (encoding hENT1) gene variations inhibit the cellular uptake of cytarabine, resulting in resistance. However, in CEM/20 X AraC cells, no *SLC29A1* variations are observed; instead, an obvious loss-of-function variation occurs in the *dCK* gene [12]. In 2017, Farge et al. reported another new mechanism of cell resistance to cytarabine. AML cells that persisted in mouse marrow after cytarabine treatment demonstrated increased oxidative phosphorylation, and inhibition of oxidative phosphorylation could restore sensitivity to cytarabine [16]. However, why increases in mitochondrial metabolism and oxidative phosphorylation can cause cells to develop resistance to cytarabine remains unclear. High-dose cytarabine-based therapy exerts the strongest antileukemic effect among AML treatments [7] and may act through another specific pathway. The considerable increase in the AMP/ATP ratio induced by high-dose cytarabine can trigger AMP-activated protein kinase (AMPK) and subsequently forkhead box, class O to promote cell cycle arrest. Furthermore, the considerable decrease in the CDP pool induced by high dose cytarabine may accelerate the reduction of dCTP, thereby increasing DNA synthesis disturbance [7]. Until now, no therapeutic strategies have been developed that prevent or overcome cytarabine resistance. 

The main objectives of this study were to comprehensively investigate the mechanism of cytarabine resistance using the gene expression assay and identify the method to overcome cytarabine resistance. This study revealed that NF-κB pathway is the top signaling pathway with the most differentially expressed genes (DEGs) during the development of R-HL60 cells. In addition, we demonstrated that proteasome inhibitors may be useful as a therapeutic agent in R-HL60 cells because of their capacity to inactivate the NF-kB pathway and induce cell death.

## 2. Results

### 2.1. Different Gene Expression Profiles between Parental HL60 and R-HL60 Cells

**Figure 1:**
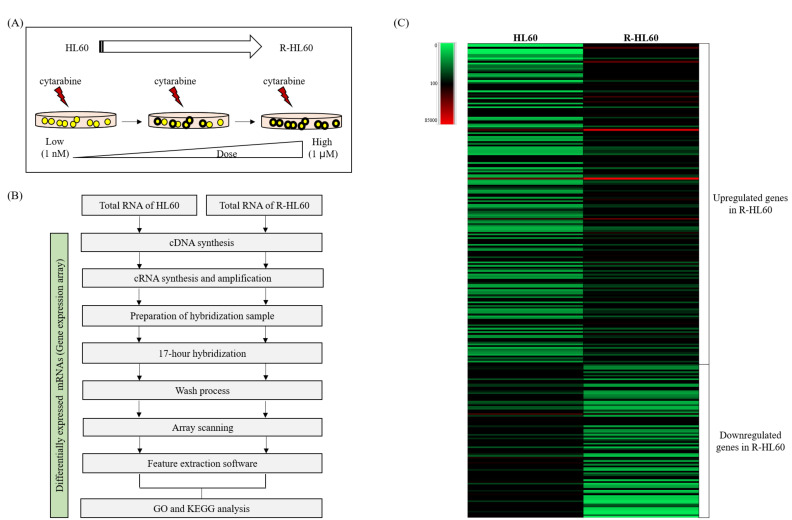
Results of the gene expression array procedures. (**A**) The R-HL60 cell line was established through continual treatment of parental HL60 cells with increasing concentrations of cytarabine. (**B**) The experimental flow chart of the gene expression array. (**C**) Heat map depicting the hierarchical clustering of changed differentially expressed genes (DEGs) between HL60 and R-HL60 cells. In the clustering analysis, upregulated and downregulated genes are colored in red and green, respectively.

A schematic of the establishment process of R-HL60 cells is depicted in Figure 1A. Total RNA from HL60 and R-HL60 cells was extracted and used for gene expression analysis. The experimental flow chart of the gene expression array was shown in Figure 1B. The heat map presents the hierarchical clustering of changed DEGs between HL60 and R-HL60 cells. In the clustering analysis, upregulated and downregulated genes are colored in red and green, respectively (Figure 1C). The results indicated a change in the gene expression profile during the development of cytarabine resistance in HL60 cells. In R-HL60 cells, 14, 40, and 131 genes exhibited over 10, 3–10, and 2–3 times higher expression, respectively, than they did in parental HL60 cells (Supplementary Table S1). Additionally, 9, 26, and 54 genes respectively exhibited 10, 3–10, and 2–3 times lower expression than they did in the parental HL60 cells (Supplementary Table S2).

### 2.2. NF-κB Pathway Was the Top Signaling Pathway with the Most DEGs during the Development of Cytarabine Resistance

**Figure 2:**
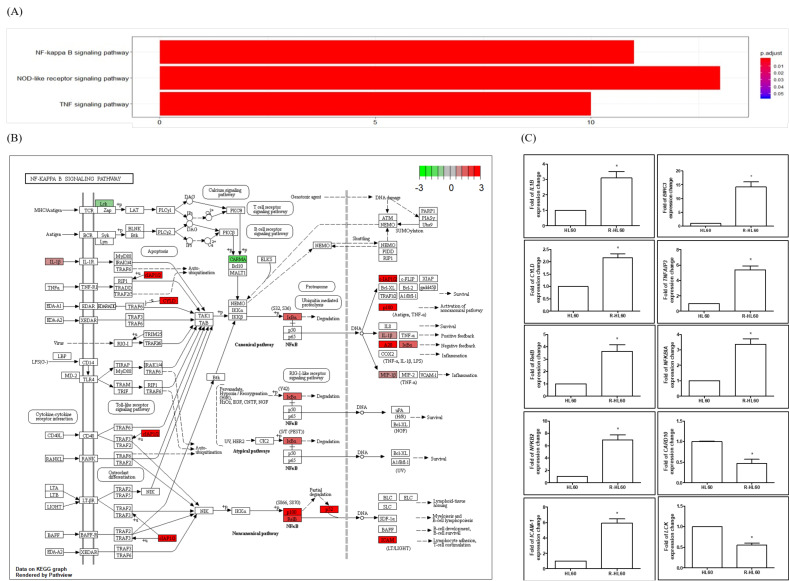
Nuclear factor κB (NF-κB) pathway is the top signaling pathway with the most DEGs during the development of R-HL60 cells. (**A**) Changes in signaling pathways during the development of parental HL60 cells into cytarabine-resistant HL60 cells were measured through KEGG pathway enrichment analysis. According to the adjusted *p* value, the greatest changes were observed in the NF-κB signaling pathway. (**B**) Map of the NF-κB signaling pathway with the lowest *p.*adjust (2.64 × 10^−6^). The expression of 11 genes (*IL1B*, *CYLD*, *RELB*, *CCL4L2*, *NFKB2*, *ICAM1*, *BIRC3*, *TNFAIP3*, *NFKBIA*, *CARD10*, and *LCK*) involved in this pathway changed significantly. The upward and downward thermometers marked in red and green indicate upregulated and downregulated signals, respectively. The solid line represents direct action, and the dashed line represents indirect action. (**C**) Gene expression was validated using real-time PCR analysis, the results of which were largely consistent with the results of the gene expression array. The expression of *CCL4L2* could not be detected because of limitations of real-time PCR analysis. The paired *t*-test was applied to determine the differences between HL60 and R-HL60 cells. The data are represented as the mean ± standard error of three independent experiments, with * *p* < 0.05 indicating a statistically significant difference. A20 (encoded by TNFAIP3); IκBα (encoded by NFKBIA); NFKB2 (encoded by p52 and p100); CARD10 (encoded by CARMA3); cIAP2 (encoded by BIRC3).

The differential gene expression of HL60 and R-HL60 cells was analyzed through Kyoto Encyclopedia of Genes and Genomes (KEGG) pathway enrichment analysis using clusterProfiler 3.14 software. After *p* adjustment, the top signaling pathway with the most DEGs was the NF-κB pathway (Figure 2A and Supplementary Figure S1). During the development of cytarabine-resistance, the expression of 11 genes involved in the NF-κB signaling pathway changed significantly. Among these, the expression of nine—namely, *IL1B*, *CYLD*, *RELB*, *CCL4L2*, *NFKB2 (p100/p52)*, *ICAM1*, *BIRC3*, *TNFAIP3*, and *NFKBIA*—increased more than two-fold, whereas the expression of *CARD10* and *LCK* genes decreased more than two-fold (Figure 2B). The changes in the expression of 11 genes associated with the NF-κB signaling pathway were verified using real-time polymerase chain reaction (PCR; Figure 2C). The results of the real-time PCR and gene expression array were a consistent trend. However, the gene sequence of *CCL4L2* (NM_001291470) is notably similar to that of *CCL4* (NM_002984.4), rendering the design of a specific primer sequence for *CCL4L2* difficult. Therefore, real-time PCR verification of *CCL4L2* was not performed. The sequences of the primers used for the real-time PCR are listed in Table 1. The results of the gene expression array and real-time PCR are presented in Table 2.

### 2.3. Proteasome Inhibitors Inhibited the Activation of Non-Canonical NF-κB Signaling Pathway in R-HL60 Cells

**Figure 3:**
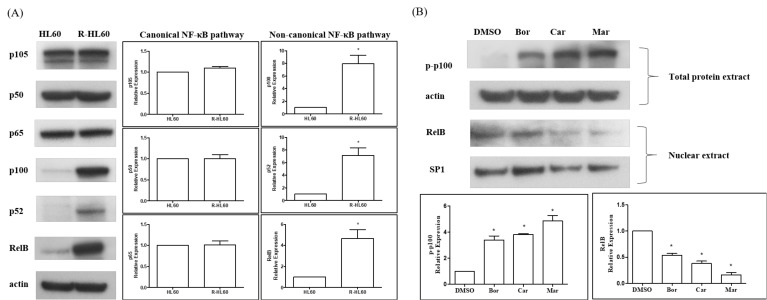
Proteasome inhibitors inhibited the activation of non-canonical NF-κB signaling pathway in R-HL60 cells. (**A**) The protein expression of NF-κB1 p105, p50, and p65 was not significantly different between HL60 and R-HL60 cells. In addition, the protein expression of NF-κB2 p100, p52, and RelB was higher in R-HL60 than in HL60 cells. The paired *t*-test was applied to determine the differences between HL60 and R-HL60 cells. The data are represented as the mean ± standard error of three independent experiments, with * *p* < 0.05 indicating a statistically significant difference. (**B**) Proteasome inhibitors (200 nM bortezomib, carfilzomib, or marizomib) induced phosphorylated p100 in the total protein extract and reduced RelB expression in the nuclear extracts after proteasome inhibitor treatment for 4 h. The one-way ANOVA was used to determine the differences between the experimental and vehicle control (dimethyl sulfoxide) group and Newman-Keuls multiple comparison test was applied as a post hoc test. The data are represented as the mean ± standard error of three independent experiments, with * *p* < 0.05 indicating a statistically significant difference. Bor: bortezomib; Car: carfilzomib; Mar: marizomib.

The expression of NF-κB subunit, including p105, p50, p65, p100, p52, and RelB was compared between HL60 and R-HL 60 cells through Western blotting. The protein expression of NF-κB1 p105, p50, and p65 was not significantly different between HL60 and R-HL60 cells, but the protein expression of NF-κB2 p100, p52, and RelB was higher in R- HL60 than in HL60 cells (Figure 3A). The ability of proteasome inhibitors to reduce the activity of non-canonical NF-κB signaling pathway was then investigated. R-HL60 cells were treated with 200 nM bortezomib, carfilzomib, or marizomib for 4 h. The phosphorylated p100 of the total protein extract and RelB of the nuclear extract were detected through Western blotting. The results revealed that bortezomib, carfilzomib, and marizomib all induced phosphorylated p100 in the total protein extract and reduced RelB expression in the nuclear extracts (Figure 3B).

### 2.4. Proteasome Inhibitors Induced Apoptosis in R-HL60 Cells

**Figure 4:**
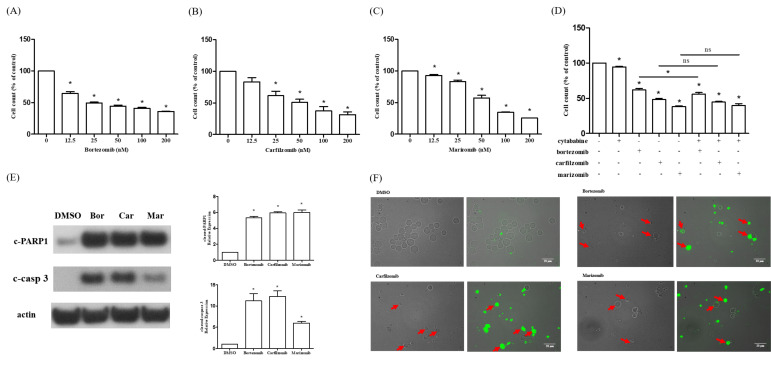
Proteasome inhibitors induce cytotoxicity in R-HL60 cells. The cytotoxic effects of (**A**) bortezomib, (**B**) carfilzomib, (**C**) marizomib, and (**D**) cytarabine combined with each proteasome inhibitor in R-HL60 cells were detected using the CCK-8 assay after proteasome inhibitors treatment for 24 h (cytarabine: 100 µM; bortezomib: 200 nM; carfilzomib: 200 nM; marizomib: 200 nM). (**E**) The levels of cleaved-PARP1 and cleaved-caspase 3 in R-HL60 cells were detected using Western blotting following proteasome inhibitor treatment for 24 h (200 nM bortezomib, carfilzomib, and marizomib). (**F**) Apoptosis cells were detected using an Apopxin Green Indicator after treatment with proteasome inhibitors for 24 h (200 nM bortezomib, carfilzomib, and marizomib). The left panel in each figure depicts an image captured using an inverted light microscope, and the right panel depicts a magnified image (400×) derived from an inverted light microscope and immunofluorescence signal (FITC channel). The one-way ANOVA was performed to determine the differences between the experimental and control group, and the Newman-Keuls multiple comparison test was applied as a post hoc test. The data are represented as the mean ± standard error of three independent experiments, with * *p* < 0.05 indicating a statistically significant difference.

**Figure 5:**
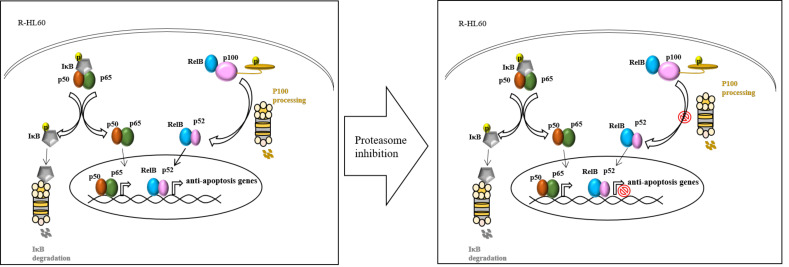
A schematic of the possible mechanism through which proteasome inhibitors induce cell apoptosis through regulation of the non-canonical NF-κB signaling pathway in R HL60 cells.

R-HL60 cells were treated with different concentrations of proteasome inhibitors, including bortezomib, carfilzomib, or marizomib for 24 h. Then, the cytotoxicity was detected using the cell counting kit-8 (CCK-8) assay. The results demonstrated that bortezomib (Figure 4A), carfilzomib (Figure 4B), and marizomib (Figure 4C) effectively reduced cell viability in a dose-dependent manner. Whether the combination of proteasome inhibitor and cytarabine increased cytotoxicity in R-HL60 was also investigated (Figure 4D). The results demonstrated that the cytotoxicity of bortezomib combined with cytarabine was significantly higher than that of bortezomib or cytarabine alone. However, most of the cytotoxicity was attributable to bortezomib. Moreover, no statistically significant difference was observed in the cytotoxicity of carfilzomib combined with cytarabine and carfilzomib alone. Similarly, the cytotoxicity of marizomib combined with cytarabine and marizomib alone was not statistically different. In addition, R-HL60 cells treated with 200 nM bortezomib, carfilzomib, or marizomib for 24 h and the expression of apoptosis protein were detected through Western blotting. The results demonstrated that bortezomib, carfilzomib, and marizomib effectively enhanced the level of cleaved-PARP1 and cleaved-caspase 3 (Figure 4E). Caspase-3 is a specific effector that is cleaved at the initiation of apoptosis and thus activated. Cleaved caspase-3 propagates apoptotic signal through enzymatic activity on downstream targets, including PARP1. PARP1 is a nuclear enzyme that is cleaved into fragments by caspases during apoptosis; thus, cleaved-PARP1 is a useful marker of apoptosis [17,18]. In addition, the apoptotic cells were detected using an Apopxin Green Indicator under a fluorescence microscope in the FITC (green) channel. The results revealed that the populations of apoptotic cells were greater in the proteasome inhibitor group (bortezomib, cartilizomib, and marizomib) than in the control group (dimethyl sulfoxide, DMSO). A schematic of the possible mechanism through which proteasome inhibitors induce cell apoptosis through the regulation of the non-canonical NF-κB signaling pathway in R-HL60 cells is presented in Figure 5.

## 3. Discussion

Differences in the gene expression profiles and pivotal pathways of parental HL60 and R-HL60 cells were investigated in this study. Our results indicate a change in the gene expression profile during the development of cytarabine resistance in HL60 cells. The changes in gene expression related to the NF-κB signaling pathway were particularly pronounced. NF-κB is a well-known family of dimeric transcription factors essential for the coordination of inflammatory response, innate and adaptive immunity, and cell differentiation, proliferation, and survival in almost all multicellular organisms [19]. Activation of NF-κB signaling promotes resistance to programmed cell death mainly through the upregulation of anti-apoptotic protein expression [20].

Cytarabine can activate NF-κB, which can induce resistance to chemotherapeutic agents. Translocation of NF-κB into the nucleus contributes to the activation of telomerase. High levels of telomerase activity are associated with treatment failure in chemo-resistant leukemia [21]. Furthermore, acid ceramidase promotes drug resistance in AML through the upregulation of NF-κB-dependent expression of the multidrug resistance 1 gene encoding P-glycoprotein. Conversely, acid ceramidase inhibitor significantly reduces cytarabine resistance in vincristine resistant HL60 cells [2]. Cyr61 is a secreted extracellular matrix protein that affects cell proliferation, survival, migration, and differentiation and drug resistance in various tumors. Exogenous Cyr61 effectively reduces cytarabine-induced apoptosis through the NF-κB signaling pathway in acute lymphoblastic leukemia cells [22]. Upregulation of NF-κB is the main molecular process driving the development of cytarabine resistance in Z138 cells [23]. Cripto-1, an oncoprotein, promotes resistance to cytarabine-induced apoptosis by activating the TAK-1/NF-κB/survivin signaling pathway [24].

Two main NF-κB activation pathways, namely the canonical and non-canonical, exist in cells [25,26]. Dysregulated NF-κB activity can cause various diseases, including cancer [19,27], and NF-κB has been regarded as a potential target for the treatment of such diseases. The NF-κB family in mammals comprises the following five members: p65 (RelA), RelB, c-Rel, NF-κB1 (p50 and its precursor p105), and NF-κB2 (p52 and its precursor p100), which can form various heterodimers and homodimers [28,29,30]. The activity of NF-κB is tightly controlled at multiple levels by positive and negative regulatory elements. The canonical pathway is induced by most physiological NF-κB stimuli, including signals from cytokine receptors (such as the TNFR and interleukin-1 receptor), antigen receptors, and pattern-recognition receptors (such as toll-like receptor 4) and is, by definition, dependent on IκB kinase β (IKKβ), nuclear factor-κB essential modifier (NEMO)-mediated phosphorylation of IκBα, and nuclear translocation of mostly p65-containing heterodimers [28]. The non-canonical NF-κB signaling pathway is induced by specific members of the TNF cytokine family, such as CD40 ligand, BAFF, and lymphotoxin-β2. Non-canonical NF-κB activation involves the phosphorylation and processing of p100. IKKα phosphorylates p100 at two C-terminal serine residues, and after ubiquitination and degradation of the ankyrin repeats, the subunit p52 is released [31,32]. The gene expression array, real-time PCR, and Western blotting performed in this study revealed no difference in the expression of p105, p50, and p65 between HL60 and R-HL60 cells; however, p100, p52, and RelB expression was upregulated in R-HL60 cells. Activation of non-canonical NF-κB signaling pathway may represent the main event in the development of cytarabine resistance.

Our results demonstrate that the upregulation of *IκBα*, *A20*, and *CYLD* and the downregulation of *LCK* and *CARD10* occur during the development of cytarabine resistance in HL60 cells. Upregulation of IκBα is the negative feedback for the canonical NF-κB pathway and can thus control canonical NF-κB signaling. Moreover, both A20 and CYLD are central negative regulators of canonical NF-κB signaling and can act by removing nonproteolytic K63-linked polyubiquitin chains from an overlapping set of signaling molecules [33,34,35]. However, in some cell systems, A20 inhibit cell apoptosis and promote cell survival, and the functions may be independent of its role in restricting NF-κB signaling [36,37]. Both LCK and CARD10 (also known as CARMA3) [38] are positive regulators of NF-κB. LCK activates NF-κB signaling through tyrosine phosphorylation of IκBα [39,40]. CARD10 is closely associated with tumor development because of its role in promoting tumor progression by activating NF-κB [41,42]. However, CARD10 has also been identified as a novel target of CEBPE, an essential transcription factor required for granulocytic differentiation [43]. In addition, posttranslational modifications, including phosphorylation, protein cleavage, glycosylation, and ubiquitination, may also support or restrict protein activity. Therefore, whether the change of these elements negatively regulate the NF-κB pathway or whether they contribute to an NF-κB-independent function are worthy of further investigation. 

Glycogen synthase kinase-3 (GSK-3) affects various signaling pathways that are crucial for cellular self-renewal, growth, and survival, including the NF-kB pathway that is critical to AML development [44,45,46]. GSK-3 can phosphorylate and stabilize S8, S17, S31, and S43 of NEMO. NEMO interacts with IKKs and is essential for NF-κB activity [46]. Studies have reported that relapse and disease progression originate from a rare population of cancer stem cells that have been observed in AML. In addition, GSK-3 mis-splicing contributes to leukemia stem cell generation [47]. Acute leukemia induced by mixed-lineage leukemia (MLL) chimeric oncoproteins belongs to a cancer subset characterized by a paradoxical dependence on GSK-3 activity for sustained proliferation [48]. GSK-3 activity is necessary for the maintenance of leukemia with MLL mutations [49]. Loss of GSK-3α induces cell differentiation in AML [50], and a GSK-3β inhibitor suppresses cell growth and induces apoptosis in leukemia cells. A study revealed that the in vivo administration of a GSK-3β inhibitor delayed tumor formation in a mouse model of leukemia [51]. Our results indicate high protein expression levels of NF-κB1 p105, p50, and p65 in both HL60 and R-HL60 cells, and these expression levels did not differ significantly between these cells. However, the protein expression levels of NF-κB2 p100, p52, and RelB were higher in the R- HL60 cells than in the HL60 cells (Figure 3A). Additionally, no significant difference in GSK-3 gene expression was observed during the development of R-HL60 cells (data not shown). The abnormal expression of GSK-3 and the activation of canonical NF-κB pathway may be implicated in AML, and the non-canonical NF-κB pathway activated through a GSK-3-independent mechanism may play a key role in the development of drug resistance.

Our results also demonstrate that proteasome inhibitors (marizomib) decreased the cell viability of parental HL60 cells (data not shown). Proteasome inhibitors may interfere with protein degradation and accumulation in the proteasome machinery. Accordingly, this finding implies that proteasome inhibitors can simultaneously kill both cytarabine-sensitive and cytarabine-resistant cells through different pathways. Proteasome inhibitors may not have preventative effects on the occurrence of cytarabine resistance but may induce the death of cytarabine resistant cells by interfering with the proteasome machinery.

HL60 cells exhibit high MYC expression as part of their ATCC-based genetic makeup. MYC promotes cell proliferation and restrains differentiation, and its transcription factor has been implicated in the control of several aspects of tumor cell biology. MYC inhibition increased the sensitivity of HL60 cells to arsenic trioxide through blunting of the PI3K/NF-κB axis [52]. Homoharringtonine (HHT) has an anti-myeloid leukemia effect and potentiates the therapeutic effectiveness of an anthracycline and cytarabine induction regimen for AML with favorable and intermediate prognoses. In addition, HHT down-regulates MYC expression by binding NF-κB repressing factor and interfering with NF-κB1 p65 translocation [53]. Whether MYC is involved in proteasome inhibitors interrupting the activation of non-canonical NF-κB signaling pathway and inducing apoptosis in R-HL60 cells warrants future investigation.

In our study, gene expression arrays and real-time PCR were used to determine the difference in gene expression between HL60 and R-HL60 cells. The gene expression array analysis results show that the expression of CYLD in R-HL60 was 85.7 times higher than that in HL60. By contrast, the real-time PCR analysis results reveal that the expression of CYLD in R-HL60 was only 2.17 times higher than that in HL60. This inconsistency may be attributed to the difference in sensitivity levels between gene expression array analysis and real-time PCR analysis. Gene expression array analysis is used for large-scale screening to reflect the overall gene expression trend of samples, but the trends of each gene may not be consistent with those identified from real-time PCR analysis. 

Studies have demonstrated that proteasome inhibition contributes to the apoptotic effect in tumor cells through the inhibition of NF-κB activity, alteration of cell cycle-related protein degradations, alteration of proapoptotic and anti-apoptotic protein balance, endoplasmic reticulum stress, and inhibition of angiogenesis and DNA repair [54,55]. The predominant biological effect of proteasome inhibitors is the inhibition of NF-κB activation. So, proteasome inhibition has become synonymous with inhibition of NF-κB [56]. In mammalian proteasomes, each β-ring contains the three catalytic β-subunits of β1, β2, and β5, which are associated with caspase-like, trypsin-like, and chymotrypsin-like activities, respectively. The active sites of these catalytic subunits face inward to receive peptide substrates from the proteasome’s hollow inner chamber. By controlling which proteins enter its inner compartment, proteasome can degrade misfolded, damaged, and short-lived proteins in a highly regulated manner [57]. Many proteasome inhibitors have been successfully used as therapeutic drugs to treat cancer. Currently, bortezomib and carfilzomib are FDA-approved proteasome inhibitors for the treatment of multiple myeloma, and marizomib is a newer drug in development [58,59]. Bortezomib is a reversible first-generation proteasome inhibitor, and carfilzomib and marizomib are irreversible second-generation proteasome inhibitors [54]. However, whether proteasome inhibitors can overcome cytarabine resistance has been rarely investigated. Our results demonstrated that bortezomib, carfilzomib, and marizomib effectively reduce the cell viability of R-HL60 cells in a dose dependent manner, inhibit the activation of non-canonical NF-κB signaling pathway, and induce apoptosis in R-HL60 cells.

However, the use of proteasome inhibitors is limited because their activity does not target NF-κB activity alone, but it also leads to the accumulation of other proteins that are degraded by the proteasome machinery. Another limitation of this study is that the effectors of the proteasome inhibitors in other resistant cell lines and clinical specimens have not been validated. This is worthy of further investigation in the future. Nevertheless, these findings provide novel therapeutic strategies for the treatment and management of relapsed or refractory leukemia.

## 4. Materials and Methods

### 4.1. Cell Culture and Drug Treatment

R-HL60 cells were established from parental HL60 cells (ATCC, Rockville, MD, USA) and were characterized as described previously [1]. HL60 and R-HL60 cells were cultured in RPMI 1640 medium (Cytiva, Marlborough, MA, USA) supplemented with 10% fetal bovine serum (Gibco, Amarillo, TX, USA) and 1% antibiotic-antimycotic reagents (Gibco). Cells were cultured at an incubator with 5% CO_2_ at a temperature of 37 °C, and the culture medium was changed every 2–3 days. Cytarabine, bortezomib, carfilzomib, and marizomib were purchased from Sigma Aldrich (St. Louis, MO, USA). R-HL60 cells were exposed to different concentrations of cytarabine, bortezomib, carfilzomib, and marizomib, and the cell viability was measured using the CCK-8 assay (Sigma Aldrich, St. Louis, MO, USA) All data were compared with the vehicle control (ddH_2_O for cytarabine treatment; dimethyl sulfoxide [DMSO] for bortezomib, carfilzomib, and marizomib treatment).

### 4.2. Gene Expression Array

RNA was extracted from parental HL60 and R- HL60 cells using TRIzol reagent (Thermo Fisher Scientific, Waltham, MA, USA) and submitted to Welgene Biotech (http://www.welgene.com.tw/, accessed on 1 November 2021) for array. Subsequently, 0.2 μg of total RNA was amplified using a Low Input Quick Amp Labeling Kit (Agilent Technologies, Santa Clara, CA, USA) and labeled with fluorescent Cy3 (Agilent Technologies) during the in vitro transcription process. Through incubation with fragmentation buffer at 60°C for 30 min, 0.6 μg of Cy3-labled cRNA was fragmented to an approximate average size of 50–100 nucleotides. Then, correspondingly fragmented and labeled cRNA was pooled and hybridized for use with a SurePrint Microarray (Agilent Technologies) at 65 °C for 17 h. The Cy3 microarray was scanned at 535 nm with a microarray scanner (Agilent Technologies) after purging with a nitrogen gun and drying. The scans were analyzed using Feature Extraction 10.7.3.1 (Agilent Technologies). The raw signal data were subjected to quantile normalization to identify differential gene expression. The DEGs were subjected to an enrichment test for functional assay, and cluster Profiler 3.14 was used for KEGG pathway analyses.

### 4.3. Real-Time Polymerase Chain Reaction

Real-time polymerase chain reaction (PCR) was conducted using Brilliant III Ultra-Fast SYBR Green QPCR Master Mix on a G8830A AriaMx Real-Time PCR System (Agilent Technologies, Santa Clara, CA, USA). The PCR program consisted of pre-incubation at 95 °C for 3 min, followed by 40 cycles of annealing/extension (95 °C for 5 s and 60 °C for 10 s). The melt curve analysis (95 °C for 30 s, 60 °C for 30 s, and 95 °C for 30 s) was performed immediately following the last extension step. Homo sapiens TATA-binding protein expression was employed as the internal control and quantified using the 2^−ΔΔCt^ method. The primer sequences are listed in Table 1.

### 4.4. Western Blotting

Cell density at 5 × 10^6^ was prepared on each 10 cm dish and cells were incubated with different drug doses for 24 h. Then, cell pellets were lysed using RIPA lysis buffer (Thermo Fisher Scientific, Waltham, MA, USA) and supplemented with the Thermo Fisher Scientific Halt Protease Inhibitor Cocktail. The samples were centrifuged at 13,000× *g* for 15 min at 4 °C; the supernatant proteins were then collected for Western blotting. Protein concentrations were measured using a Thermo Fisher Scientific Pierce BCA ProteinAssay Kit. Protein lysates (20–30 µg) were loaded onto Bolt 4–12% Bis-Tris Plus gels (Invitrogen, Waltham, MA, USA) and transferred to polyvinylidene difluoride membranes. The membranes were blocked with 5% nonfat milk in phosphate buffered saline with 0.1% Tween-20 for 1 h at room temperature. The blots were incubated overnight at 4 °C with primary antibodies, all of which had been purchased from Cell Signaling Technology (Beverly, MA, USA), except the mouse anti-β actin antibody (Novus Biologicals, Centennial, CO, USA). The primary antibodies were diluted at 1:1000, except anti-β actin antibody (1:30,000) and anti-c-PARP (1:15,000). After the primary antibodies were removed, the blots were incubated with horseradish peroxidase-linked secondary antibodies (1:10,000; GE Healthcare, Waukesha, WI, USA) for 1 h at room temperature, and reading was performed using an enhanced chemilu-minescence assay kit (Thermo Fisher Scientific).

### 4.5. Subcellular Fractionation

Subcellular fractionation was performed using a Subcellular protein fractionation kit (Thermo Fisher Scientific) and the assay was performed according to the modified manufacturer’s protocol. In brief, cells were centrifuged at 500× *g* for 5 min and then washed through the suspension of the cell pellet in ice-cold PBS. Ice-cold CEB containing protease inhibitors were added to the cell pellet and incubated in the tube at 4 °C for 3 min with gentle mixing. After centrifugation at 500× *g* for 3 min, the cytosol fraction was obtained. Then, RIPA lysis buffer containing protease inhibitors was added to the pellet and the tube was vortexed on the highest setting for 15 s. The supernatant was then incubated in the tube at 4 °C for 30 min with gentle mixing. After centrifugation at 13,000× *g* for 20 min, the supernatant (nuclear fraction) was immediately transferred to a clean ice-chilled tube.

### 4.6. Cell Viability Assay

The CCK-8 assay was conducted in R-HL60 cells to measure the cytotoxic effect of bortezomib, carfilzomib, and marizomib. R-HL60 cells were seeded at a concentration of 5 × 10^4^ cells/mL into a 96-well plate. Following overnight adherence, the cells were treated with 0–200 nM bortezomib, carfilzomib, and marizomib (dissolved in DMSO to produce a stock solution) for 24 h. Then, 10 μL CCK-8 solution was added to each well and the plate was incubated at 37 °C for 2 h. Specific absorbance and reference absorbance were measured at 450 and 650 nm, respectively, on an enzyme-linked immunosorbent assay reader.

### 4.7. Immunofluorescence and Microscopy Examination

After treatment, the culture medium was discarded, and the cells were washed twice with 200 μL of Assay Buffer. Subsequently, the cells were incubated in a mixture of 200 μL of Assay Buffer and 2 μL of an Apopxin Green Indicator (#ab176749; Abcam, Cambridge, UK) at room temperature for 60 min. Immunofluorescence images were obtained using a microscope (DMI6000; Leica Microsystems, Heidelberg, Germany).

### 4.8. Statistical Analysis

The data were analyzed using GraphPad Prism5.0 (GraphPad Software Inc., San Diego, CA, USA). The paired t-tests and one-way ANOVA were used to determine the differences between the experimental and control groups and Newman-Keuls multiple comparison test was applied as a post hoc test. Data are represented as mean ± standard error of three independent experiments, with *p* < 0.05 indicating a statistically significant difference in all comparisons of the experimental and control group.

## 5. Conclusions

At present, no method has been developed to overcome drug resistance in leukemia chemotherapy. Constitutive NF-κB has been detected in 40% of patients with AML, and its aberrant activity enable leukemia cells to escape apoptosis and stimulate proliferation. Clinical trials of FDA approved proteasome inhibitors, which are also NF-κB inhibitors, in combination with other therapeutic drugs for the treatment of patients with AML are ongoing [60,61,62,63]. In cytarabine-resistant cells, the pivotal pathway that assists with cancer cell survival has not been investigated in detail. Our study is the first to report that proteasome inhibitors may overcome cytarabine resistance in patients with relapsed or refractory leukemia through the inhibition of non-canonical NF-κB pathway activation.

## Figures and Tables

**Table 1:** Sequences of primers used for real-time PCR.

Gene Symbol	Accession Number	Forward Primer (5′-3′)	Reverse Primer (5′-3′)
*IL1B*	NM_000576	TGATGAGCAACCGCTTCCC	AAAAACTAGGCTCTTTTACAGACAC
*CYLD*	NM_015247	TGTGAAGTATGGGAAGGACGA	TCTCCTACTTCTGGGCATGG
*RELB*	NM_006509	CGAGAAGCTCCGTTGCAC	AATCCTACTGGAGAAGGTGCCC
*NFKB2*	NM_001288724	ACTGTCACTTGGTGATACAGCTC	TACGTGTCTACCAGGCTGCG
*ICAM1*	NM_000201	TGCTGCCTATTGGGTATGCT	GGGTTGGGGTCAGTAGACAG
*BIRC3*	NM_001165	GCCCCACCTATTGGAAGAAG	CCCAAGCATTGCTAACCAGT
*TNFAIP3*	NM_006290	TGTTAATGCCTCTGAGTGTCCT	CCTGTGACCATTGCCAGTCTC
*NFKBIA*	NM_020529	TGTGCTTCGAGTGACTGACC	TCACCCCACATCACTGAACG
*CARD10*	NM_014550	CTAACACGTGTGCGTTCCTG	ATCCACGGGCCGTACATTC
*LCK*	NM_005356	GATCCTGCTGACGGAAATTG	CAGGTTCTGAATCACCTCCG

**Table 2:** Gene expression differences determined using gene expression array and real-time PCR.

Gene Name	Fold Change (R-HL60/HL60)		
	Array	Real-Time PCR	
	Ratio	Ratio	*p*-Value
*IL1B*	2.05	3.09	0.0353
*CYLD*	85.70	2.17	0.0175
*RELB*	4.32	3.62	0.0396
*CCL4L2*	2.20	N/A	
*NFKB2*	6.92	6.94	0.0174
*ICAM1*	5.83	5.92	0.0124
*BIRC3*	17.74	14.23	0.0183
*TNFAIP3*	5.53	5.35	0.015
*NFKBIA*	2.47	3.35	0.0237
*CARD10*	0.30	0.47	0.0326
*LCK*	0.49	0.55	0.0097

N/A: Not Available.

## Supplementary Materials

The following are available online at https://www.mdpi.com/article/10.3390/ijms23010361/s1.
