# Supplementary material for: Proteasome Inhibitors Interrupt the Activation of Non-Canonical NF-κB Signaling Pathway and Induce Cell Apoptosis in Cytarabine-Resistant HL60 Cells
Source: Int J Mol Sci. 2021 Dec 29;23(1):361. doi: 10.3390/ijms23010361 (PMC8745175; doi:10.3390/ijms23010361)
Supplement: Supplementary file 1 [file ijms-23-00361-s001.zip › ijms-1483660-supplementary.pdf]

International Journal of
Molecular Sciences

MDPI

Supplementary materials

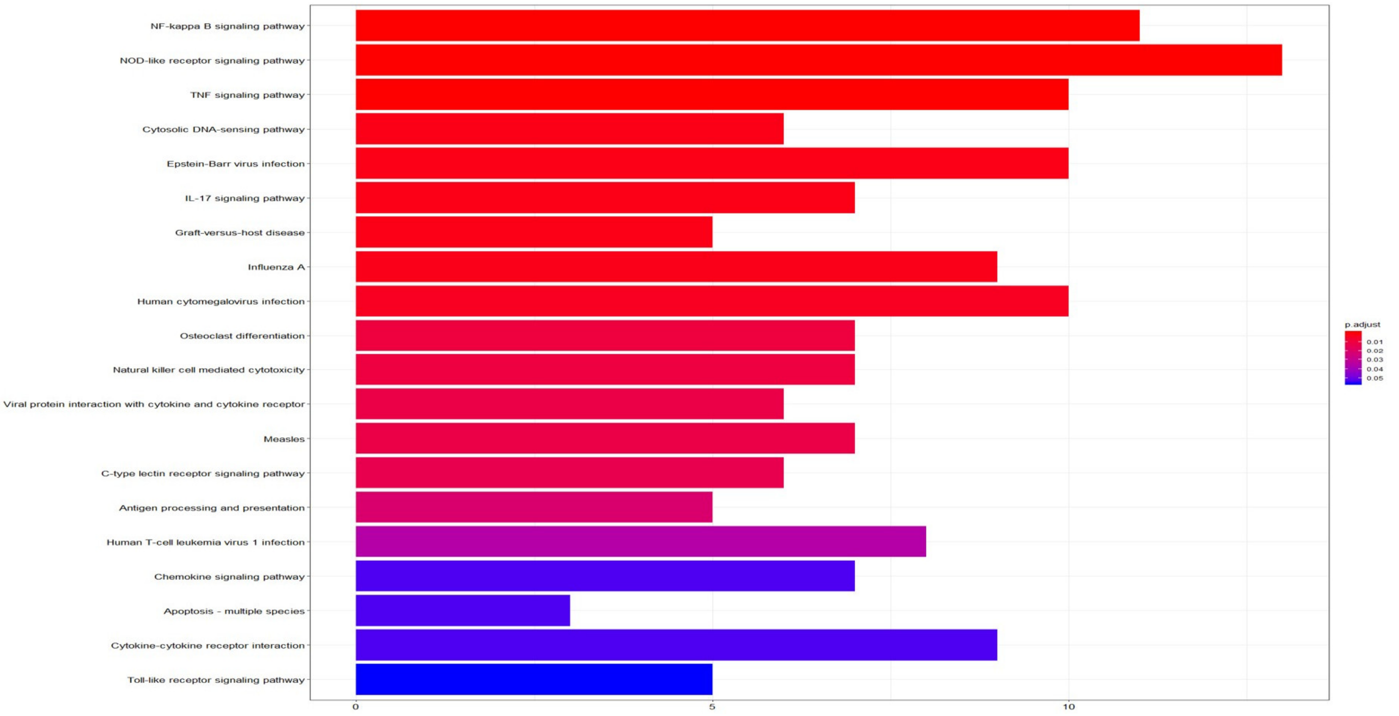

Supplementary Figure S1. Differences in signaling pathways between parental HL60 cells and R-HL60 cells. Top 20 signaling pathways ranked by the differential gene expression scores as measured using KEGG pathway analysis software. Top 1 signaling pathways were NF-kappa B signaling pathway.

Supplementary Table S1. The representative genes that is higher expression in cytarabine-resistant HL60 cells than in parental HL60 cells.

Fold Change	Gene Name	Description
225.94	CXCL10	ref|Homo sapiens chemokine (C-X-C motif) ligand 10 (CXCL10), mRNA [NM_001565]
170.24	LINC01446	ref|Homo sapiens long intergenic non-protein coding RNA 1446 (LINC01446), long non-coding RNA [NR_038371]
85.70	CYLD	ref|Homo sapiens cylindromatosis (turban tumor syndrome) (CYLD), transcript variant 1, mRNA [NM_015247]
69.77	CLPSL1	ref|Homo sapiens colipase-like 1 (CLPSL1), mRNA [NM_001010886]
47.58	lnc-ZNF623-1	linc|LNCipedia lincRNA (lnc-ZNF623-1), lincRNA [lnc-ZNF623-1:1]
40.81	LOC102724332	ens|immunoglobulin kappa constant [Source:HGNC Symbol;Acc:HGNC:5716] [ENST00000390237]
19.35	DEFA9P	ref|Homo sapiens defensin, alpha 9 pseudogene (DEFA9P), non-coding RNA [NR_073408]
17.74	BIRC3	ref|Homo sapiens baculoviral IAP repeat containing 3 (BIRC3), transcript variant 1, mRNA [NM_001165]
17.13	N4BP3	ref|Homo sapiens NEDD4 binding protein 3 (N4BP3), mRNA [NM_015111]
14.10	MIR146A	ens|microRNA 146a [Source:HGNC Symbol;Acc:HGNC:31533] [ENST00000517927]
11.63	CD70	ref|Homo sapiens CD70 molecule (CD70), mRNA [NM_001252]
11.44	ENST00000582921	Unknown
10.50	ENST00000520370	Unknown
10.34	lnc-AF127577.1-4	linc|LNCipedia lincRNA (lnc-AF127577.1-4), lincRNA [lnc-AF127577.1-4:1]
9.65	IL4I1	ref|Homo sapiens interleukin 4 induced 1 (IL4I1), transcript variant 1, mRNA [NM_152899]
9.53	ENST00000520881	Unknown
7.67	AIM2	ref|Homo sapiens absent in melanoma 2 (AIM2), mRNA [NM_004833]
7.42	LHX2	ref|Homo sapiens LIM homeobox 2 (LHX2), mRNA [NM_004789]

7.12	LINC00599	ref|Homo sapiens long intergenic non-protein coding RNA 599 (LINC00599), long non-coding RNA [NR_024281]
6.92	NFKB2	ref|Homo sapiens nuclear factor of kappa light polypeptide gene enhancer in B-cells 2 (p49/p100) (NFKB2), transcript variant 3, mRNA [NM_001288724]
6.24	BCL3	ref|Homo sapiens B-cell CLL/lymphoma 3 (BCL3), mRNA [NM_005178]
5.97	lnc-ABI1-4	linc|LNCipedia lincRNA (lnc-ABI1-4), lincRNA [lnc-ABI1-4:1]
5.83	ICAM1	ref|Homo sapiens intercellular adhesion molecule 1 (ICAM1), mRNA [NM_000201]
5.53	TNFAIP3	ref|Homo sapiens tumor necrosis factor, alpha-induced protein 3 (TNFAIP3), transcript variant 3, mRNA [NM_006290]
5.52	ASB2	ref|Homo sapiens ankyrin repeat and SOCS box containing 2 (ASB2), transcript variant 2, mRNA [NM_016150]
5.45	GHRL	ref|Homo sapiens ghrelin/obestatin prepropeptide (GHRL), transcript variant 1, mRNA [NM_016362]
5.35	CD48	ref|Homo sapiens CD48 molecule (CD48), transcript variant 1, mRNA [NM_001778]
4.99	SRP14-AS1	ref|Homo sapiens SRP14 antisense RNA1 (head to head) (SRP14-AS1), transcript variant 4, long non-coding RNA [NR_040062]
4.97	DEFA8P	ref|Homo sapiens defensin, alpha 8 pseudogene (DEFA8P), non-coding RNA [NR_073407]
4.76	CYBB	ref|Homo sapiens cytochrome b-245, beta polypeptide (CYBB), mRNA [NM_000397]
4.32	RELB	ref|Homo sapiens v-rel avian reticuloendotheliosis viral oncogene homolog B (RELB), mRNA [NM_006509]
3.89	ENST00000616656	ens|BCL2/adenovirus E1B 19kDa interacting protein 3 pseudogene 41 [Source:HGNC Symbol;Acc:HGNC:49721] [ENST00000616656]
3.86	GPR68	ref|Homo sapiens G protein-coupled receptor 68 (GPR68), transcript variant 2, mRNA [NM_003485]
3.82	CCL5	ref|Homo sapiens chemokine (C-C motif) ligand 5 (CCL5), transcript variant 1, mRNA [NM_002985]
3.77	lnc-SLAMF7-2	gb|603082601F1 NIH_MGC_120 Homo sapiens cDNA clone IMAGE:5221536 5', mRNA sequence [BI832795]
3.64	MGAT3	ref|Homo sapiens mannosyl (beta-1,4-)-glycoprotein beta-1,4-N-acetylglucosaminyltransferase (MGAT3), transcript variant 1, mRNA [NM_002409]

3.62	ENST00000417800	Unknown
3.55	IFIH1	ref|Homo sapiens interferon induced with helicase C domain 1 (IFIH1), mRNA [NM_022168]
3.45	SLC8A1	ref|Homo sapiens solute carrier family 8 (sodium/calcium exchanger), member 1 (SLC8A1), transcript variant A, mRNA [NM_021097]
3.44	TTN-AS1	ref|Homo sapiens TTN antisense RNA 1 (TTN-AS1), transcript variant 1, long non-coding RNA [NR_038272]
3.35	MMP9	ref|Homo sapiens matrix metallopeptidase 9 (gelatinase B, 92kDa gelatinase, 92kDa type IV collagenase) (MMP9), mRNA [NM_004994]
3.32	C1orf186	ref|Homo sapiens chromosome 1 open reading frame 186 (C1orf186), mRNA [NM_001007544]
3.30	SOX9	ref|Homo sapiens SRY (sex determining region Y)-box 9 (SOX9), mRNA [NM_000346]
3.25	SVIP	ref|Homo sapiens small VCP/p97-interacting protein (SVIP), mRNA [NM_148893]
3.22	COL9A2	ref|Homo sapiens collagen, type IX, alpha 2 (COL9A2), mRNA [NM_001852]
3.19	ZC3H12A	ref|Homo sapiens zinc finger CCCH-type containing 12A (ZC3H12A), mRNA [NM_025079]
3.17	lnc-C9orf50-2	linc|LNCipedia lincRNA (lnc-C9orf50-2), lincRNA [lnc-C9orf50-2:1]
3.17	XLOC_l2_006584	linc|BROAD Institute lincRNA (XLOC_l2_006584), lincRNA [TCONS_l2_00012283]
3.16	HLA-J	ens|major histocompatibility complex, class I, J (pseudogene) [Source:HGNC Symbol;Acc:HGNC:4967] [ENST00000490720]
3.16	XLOC_l2_012953	linc|BROAD Institute lincRNA (XLOC_l2_012953), lincRNA [TCONS_l2_00024711]
3.09	CD83	ref|Homo sapiens CD83 molecule (CD83), transcript variant 1, mRNA [NM_004233]
3.06	LOC731424	ref|Homo sapiens uncharacterized LOC731424 (LOC731424), long non-coding RNA [NR_037867]
3.05	UBE2L6	ref|Homo sapiens ubiquitin-conjugating enzyme E2L 6 (UBE2L6), transcript variant 2, mRNA [NM_198183]
3.00	EXOC3L4	ref|Homo sapiens exocyst complex component 3-like 4 (EXOC3L4), mRNA [NM_001077594]
2.97	LINC01559	ref|Homo sapiens long intergenic non-protein coding RNA 1559 (LINC01559), long non-coding RNA [NR_036555]

2.95	FAM155B	ref|Homo sapiens family with sequence similarity 155, member B (FAM155B), mRNA [NM_015686]
2.84	OLIG1	ref|Homo sapiens oligodendrocyte transcription factor 1 (OLIG1), mRNA [NM_138983]
2.83	FCHSD1	ref|Homo sapiens FCH and double SH3 domains 1 (FCHSD1), mRNA [NM_033449]
2.78	CD38	ref|Homo sapiens CD38 molecule (CD38), mRNA [NM_001775]
2.76	GTSCR1	ref|Homo sapiens Gilles de la Tourette syndrome chromosome region, candidate 1 (non-protein coding) (GTSCR1), mRNA [NM_001278515]
2.76	LOC102724020	ref|Homo sapiens uncharacterized LOC102724020 (LOC102724020), long non-coding RNA [NR_120484]
2.76	KLK3	ens|kallikrein-related peptidase 3 [Source:HGNC Symbol;Acc:HGNC:6364] [ENST00000595151]
2.76	XLOC_l2_009811	linc|BROAD Institute lincRNA (XLOC_l2_009811), lincRNA [TCONS_l2_00018728]
2.76	A_33_P3218491	Unknown
2.75	S100A9	ref|Homo sapiens S100 calcium binding protein A9 (S100A9), mRNA [NM_002965]
2.75	PTX3	ref|Homo sapiens pentraxin 3, long (PTX3), mRNA [NM_002852]
2.74	FCAR	ref|Homo sapiens Fc fragment of IgA, receptor for (FCAR), transcript variant 1, mRNA [NM_002000]
2.71	SERPINA1	ref|Homo sapiens serpin peptidase inhibitor, clade A (alpha-1 antiproteinase, antitrypsin), member 1 (SERPINA1), transcript variant 2, mRNA [NM_001002236]
2.69	XLOC_l2_001066	linc|BROAD Institute lincRNA (XLOC_l2_001066), lincRNA [TCONS_l2_00001463]
2.68	S1PR2	ref|Homo sapiens sphingosine-1-phosphate receptor 2 (S1PR2), mRNA [NM_004230]
2.68	RAB7B	ref|Homo sapiens RAB7B, member RAS oncogene family (RAB7B), transcript variant 1, mRNA [NM_177403]
2.67	CCL4L2	ref|Homo sapiens chemokine (C-C motif) ligand 4-like 2 (CCL4L2), transcript variant CCL4L2b2, mRNA [NM_001291470]
2.66	CRHR2	ref|Homo sapiens corticotropin releasing hormone receptor 2 (CRHR2), transcript variant 1, mRNA [NM_001883]
2.65	SMIM2	ref|Homo sapiens small integral membrane protein 2 (SMIM2), mRNA [NM_024058]

2.62	LRG1	ref|Homo sapiens leucine-rich alpha-2-glycoprotein 1 (LRG1), mRNA [NM_052972]
2.62	LOC388942	Unknown
2.59	lnc-FAM13B-1	gb|BX325639 Homo sapiens NEUROBLASTOMA COT 25-NORMALIZED Homo sapiens cDNA clone CS0DC017YN02 5-PRIME, mRNA sequence [BX325639]
2.58	HLA-B	ref|Homo sapiens major histocompatibility complex, class I, B (HLA-B), mRNA [NM_005514]
2.58	MIR100HG	ref|Homo sapiens mir-100-let-7a-2 cluster host gene (non-protein coding) (MIR100HG), long non-coding RNA [NR_024430]
2.57	LOC101928067	ens|long intergenic non-protein coding RNA 349 [Source:HGNC Symbol;Acc:HGNC:42667] [ENST00000448748]
2.57	LINC01091	ref|Homo sapiens long intergenic non-protein coding RNA 1091 (LINC01091), transcript variant 2, long non-coding RNA [NR_027106]
2.56	OAS3	ref|Homo sapiens 2'-5'-oligoadenylate synthetase 3, 100kDa (OAS3), mRNA [NM_006187]
2.55	LOC101929709	ref|Homo sapiens uncharacterized LOC101929709 (LOC101929709), long non-coding RNA [NR_125822]
2.54	SNX20	ref|Homo sapiens sorting nexin 20 (SNX20), transcript variant 1, mRNA [NM_182854]
2.53	PALLD	ens|palladin, cytoskeletal associated protein [Source:HGNC Symbol;Acc:HGNC:17068] [ENST00000508898]
2.52	MIR1247	ens|DIO3 opposite strand/antisense RNA (head to head) [Source:HGNC Symbol;Acc:HGNC:20348] [ENST00000555882]
2.52	CCL2	ref|Homo sapiens chemokine (C-C motif) ligand 2 (CCL2), mRNA [NM_002982]
2.49	LOC100506737	ref|PREDICTED: Homo sapiens uncharacterized LOC100506737 (LOC100506737), ncRNA [XR_172388]
2.47	TTN	ref|Homo sapiens titin (TTN), transcript variant N2-A, mRNA [NM_133378]
2.47	NFKBIA	ref|Homo sapiens nuclear factor of kappa light polypeptide gene enhancer in B-cells inhibitor, alpha (NFKBIA), mRNA [NM_020529]
2.47	XLOC_l2_005553	linc|BROAD Institute lincRNA (XLOC_l2_005553), lincRNA [TCONS_l2_00010286]

2.45	LINC00908	ref|Homo sapiens long intergenic non-protein coding RNA 908 (LINC00908), long non-coding RNA [NR_015417]
2.45	SPTBN1	ref|Homo sapiens spectrin, beta, non-erythrocytic 1 (SPTBN1), transcript variant 1, mRNA [NM_003128]
2.43	TBX5-AS1	ref|Homo sapiens TBX5 antisense RNA 1 (TBX5-AS1), long non-coding RNA [NR_038440]
2.39	lnc-OLFM3-1	linc|LNCipedia lincRNA (lnc-OLFM3-1), lincRNA [lnc-OLFM3-1:3]
2.38	TMEM92-AS1	ref|Homo sapiens TMEM92 antisense RNA 1 (TMEM92-AS1), long non-coding RNA [NR_125805]
2.35	TUBB4A	ref|Homo sapiens tubulin, beta 4A class IVa (TUBB4A), transcript variant 3, mRNA [NM_006087]
2.35	OR4A15	ref|Homo sapiens olfactory receptor, family 4, subfamily A, member 15 (OR4A15), mRNA [NM_001005275]
2.34	PRR27	ref|Homo sapiens proline rich 27 (PRR27), mRNA [NM_214711]
2.33	lnc-SLA2-2	linc|LNCipedia lincRNA (lnc-SLA2-2), lincRNA [lnc-SLA2-2:1]
2.32	HLA-F	ref|Homo sapiens major histocompatibility complex, class I, F (HLA-F), transcript variant 2, mRNA [NM_018950]
2.32	CARD17	ref|Homo sapiens caspase recruitment domain family, member 17 (CARD17), mRNA [NM_001007232]
2.31	GNGT2	ref|Homo sapiens guanine nucleotide binding protein (G protein), gamma transducing activity polypeptide 2 (GNGT2), transcript variant 1, mRNA [NM_031498]
2.31	lnc-C1orf106-1	gb|Homo sapiens cDNA FLJ12831 fis, clone NT2RP2003099. [AK022893]
2.30	A_33_P3340189	Unknown
2.30	TAGLN3	ref|Homo sapiens transgelin 3 (TAGLN3), transcript variant 1, mRNA [NM_013259]
2.30	LOC100507530	ref|Homo sapiens uncharacterized LOC100507530 (LOC100507530), long non-coding RNA [NR_125386]
2.30	OR4X2	ref|Homo sapiens olfactory receptor, family 4, subfamily X, member 2 (gene/pseudogene) (OR4X2), mRNA [NM_001004727]
2.30	A_33_P3319502	Unknown
2.30	lnc-SNRPD3-2	linc|LNCipedia lincRNA (lnc-SNRPD3-2), lincRNA [lnc-SNRPD3-2:1]
2.29	DDIT3	ref|Homo sapiens DNA-damage-inducible transcript 3 (DDIT3), transcript variant 5, mRNA [NM_004083]

2.29	lnc-ASB4-2	linc|LNCipedia lincRNA (lnc-ASB4-2), lincRNA [lnc-ASB4-2:1]
2.28	PSMB9	ref|Homo sapiens proteasome (prosome, macropain) subunit, beta type, 9 (PSMB9), mRNA [NM_002800]
2.26	IL10RA	ref|Homo sapiens interleukin 10 receptor, alpha (IL10RA), transcript variant 1, mRNA [NM_001558]
2.26	lnc-SMC1B-3	linc|LNCipedia lincRNA (lnc-SMC1B-3), lincRNA [lnc-SMC1B-3:1]
2.26	lnc-NRSN1-3	linc|LNCipedia lincRNA (lnc-NRSN1-3), lincRNA [lnc-NRSN1-3:2]
2.26	ARL4C	ref|Homo sapiens ADP-ribosylation factor-like 4C (ARL4C), transcript variant 1, mRNA [NM_001282431]
2.26	ENST00000556773	Unknown
2.24	lnc-LYSMD2-1	linc|LNCipedia lincRNA (lnc-LYSMD2-1), lincRNA [lnc-LYSMD2-1:1]
2.24	ADAM8	ref|Homo sapiens ADAM metallopeptidase domain 8 (ADAM8), transcript variant 1, mRNA [NM_001109]
2.22	lnc-NLGN2-1	linc|LNCipedia lincRNA (lnc-NLGN2-1), lincRNA [lnc-NLGN2-1:1]
2.20	PTGER4	ref|Homo sapiens prostaglandin E receptor 4 (subtype EP4) (PTGER4), mRNA [NM_000958]
2.19	RBM14	gb|Homo sapiens mRNA for RNA binding motif protein 14 variant protein. [AB209007]
2.18	FAM49A	ref|Homo sapiens family with sequence similarity 49, member A (FAM49A), mRNA [NM_030797]
2.17	BBC3	ref|Homo sapiens BCL2 binding component 3 (BBC3), transcript variant 4, mRNA [NM_014417]
2.16	LOC102724463	ref|PREDICTED: Homo sapiens uncharacterized LOC102724463 (LOC102724463), ncRNA [XR_425971]
2.15	lnc-HEPH-1	gb|Homo sapiens mir-223 transcript variant 1 mRNA, complete sequence. [DQ680071]
2.15	GPR142	ref|Homo sapiens G protein-coupled receptor 142 (GPR142), mRNA [NM_181790]
2.15	GAS7	ref|Homo sapiens growth arrest-specific 7 (GAS7), transcript variant c, mRNA [NM_201433]
2.14	lnc-CHADL-1	gb|Homo sapiens cDNA FLJ32615 fis, clone STOMA2000148. [AK057177]
2.13	ACER2	ref|Homo sapiens alkaline ceramidase 2 (ACER2), mRNA [NM_001010887]
2.13	TRIM22	ref|Homo sapiens tripartite motif containing 22 (TRIM22), transcript variant 1, mRNA [NM_006074]
2.13	BMF	ref|Homo sapiens Bcl2 modifying factor (BMF), transcript variant 1, mRNA [NM_001003940]

2.12	PDK4	ref|Homo sapiens pyruvate dehydrogenase kinase, isozyme 4 (PDK4), mRNA [NM_002612]
2.12	lnc-GABPA-5	linc|LNCipedia lincRNA (lnc-GABPA-5), lincRNA [lnc-GABPA-5:1]
2.12	ABI3	ref|Homo sapiens ABI family, member 3 (ABI3), transcript variant 1, mRNA [NM_016428]
2.12	PTGIR	ref|Homo sapiens prostaglandin I2 (prostacyclin) receptor (IP) (PTGIR), mRNA [NM_000960]
2.11	LOC727721	gb|DB238770 TRACH3 Homo sapiens cDNA clone TRACH3034794 5', mRNA sequence [DB238770]
2.11	ANO7	ref|Homo sapiens anoctamin 7 (ANO7), transcript variant NGEP-L, mRNA [NM_001001891]
2.10	TCF23	ref|Homo sapiens transcription factor 23 (TCF23), mRNA [NM_175769]
2.10	ATF3	ref|Homo sapiens activating transcription factor 3 (ATF3), transcript variant 4, mRNA [NM_001040619]
2.10	IER3	ref|Homo sapiens immediate early response 3 (IER3), mRNA [NM_003897]
2.10	BG205415	gb|RST24971 Athersys RAGE Library Homo sapiens cDNA, mRNA sequence [BG205415]
2.10	LAX1	ref|Homo sapiens lymphocyte transmembrane adaptor 1 (LAX1), transcript variant 1, mRNA [NM_017773]
2.10	lnc-AC099552.4.1-1	linc|LNCipedia lincRNA (lnc-AC099552.4.1-1), lincRNA [lnc-AC099552.4.1-1:2]
2.09	AOX1	ref|Homo sapiens aldehyde oxidase 1 (AOX1), mRNA [NM_001159]
2.09	ALOX5AP	ref|Homo sapiens arachidonate 5-lipoxygenase-activating protein (ALOX5AP), transcript variant 1, mRNA [NM_001629]
2.09	CARD16	ref|Homo sapiens caspase recruitment domain family, member 16 (CARD16), transcript variant 1, mRNA [NM_001017534]
2.09	lnc-WNT1-3	linc|LNCipedia lincRNA (lnc-WNT1-3), lincRNA [lnc-WNT1-3:1]
2.09	IL15RA	ref|Homo sapiens interleukin 15 receptor, alpha (IL15RA), transcript variant 2, mRNA [NM_172200]
2.09	THC2739440	tc|BC020650 SEC3L1 protein {Homo sapiens} (exp=-1; wgp=0; cg=0), partial (18%) [THC2739440]
2.08	LOC101929759	ref|PREDICTED: Homo sapiens uncharacterized LOC101929759 (LOC101929759), ncRNA [XR_242480]

2.08	A_33_P3216200	Unknown
2.08	LOC340357	ref|Homo sapiens uncharacterized LOC340357 (LOC340357), long non-coding RNA [NR_015383]
2.07	AADACL2-AS1	ref|Homo sapiens AADACL2 antisense RNA 1 (AADACL2-AS1), transcript variant 2, long non-coding RNA [NR_110203]
2.07	SMIM2-AS1	ref|Homo sapiens SMIM2 antisense RNA 1 (SMIM2-AS1), transcript variant 1, long non-coding RNA [NR_104064]
2.07	lnc-DAAM2-3	tc|Q3Y452_HUMAN (Q3Y452) Testis development related protein 1, complete [THC2717314]
2.07	CRNN	ref|Homo sapiens cornulin (CRNN), mRNA [NM_016190]
2.07	GBP3	ref|Homo sapiens guanylate binding protein 3 (GBP3), mRNA [NM_018284]
2.07	DAPP1	ref|Homo sapiens dual adaptor of phosphotyrosine and 3-phosphoinositides (DAPP1), mRNA [NM_014395]
2.06	PMAIP1	ref|Homo sapiens phorbol-12-myristate-13-acetate-induced protein 1 (PMAIP1), mRNA [NM_021127]
2.06	ENST00000445613	Unknown
2.06	CLEC9A	ref|Homo sapiens C-type lectin domain family 9, member A (CLEC9A), mRNA [NM_207345]
2.05	ENST00000612330	gb|Homo sapiens cDNA FLJ35556 fis, clone SPLEN2004844. [AK092875]
2.05	GPR132	ref|Homo sapiens G protein-coupled receptor 132 (GPR132), transcript variant 2, mRNA [NM_013345]
2.05	lnc-GABRB3-1	linc|LNCipedia lincRNA (lnc-GABRB3-1), lincRNA [lnc-GABRB3-1:1]
2.05	IL1B	ref|Homo sapiens interleukin 1, beta (IL1B), mRNA [NM_000576]
2.04	TMEM37	ref|Homo sapiens transmembrane protein 37 (TMEM37), mRNA [NM_183240]
2.04	lnc-NDE1-2	gb|DA304148 BRHIP2 Homo sapiens cDNA clone BRHIP2019949 5', mRNA sequence [DA304148]
2.04	PTGS1	ref|Homo sapiens prostaglandin-endoperoxide synthase 1 (prostaglandin G/H synthase and cyclooxygenase) (PTGS1), transcript variant 1, mRNA [NM_000962]
2.03	LINC01215	ref|Homo sapiens long intergenic non-protein coding RNA 1215 (LINC01215), transcript variant 1, long non-coding RNA [NR_110028]

2.03	FOLR2	ref|Homo sapiens folate receptor 2 (fetal) (FOLR2), transcript variant 1, mRNA [NM_000803]
2.03	THC2710983	tc|NUD16_HUMAN (Q96DE0) Nucleoside diphosphate-linked moiety X motif 16 (Nudix motif 16) , partial (70%) [THC2710983]
2.03	CPS1	ref|Homo sapiens carbamoyl-phosphate synthase 1, mitochondrial (CPS1), transcript variant 2, mRNA [NM_001875]
2.02	lnc-FAM82A1-1	linc|LNCipedia lincRNA (lnc-FAM82A1-1), lincRNA [lnc-FAM82A1-1:1]
2.02	LOC100289230	ref|Homo sapiens uncharacterized LOC100289230 (LOC100289230), long non-coding RNA [NR_036530]
2.01	OAS2	ref|Homo sapiens 2'-5'-oligoadenylate synthetase 2, 69/71kDa (OAS2), transcript variant 1, mRNA [NM_016817]
2.01	CRYM	ref|Homo sapiens crystallin, mu (CRYM), mRNA [NM_001888]
2.01	lnc-C3orf71-1	linc|LNCipedia lincRNA (lnc-C3orf71-1), lincRNA [lnc-C3orf71-1:2]
2.01	lnc-MAGEA4-1	gb|601440791F1 NIH_MGC_72 Homo sapiens cDNA clone IMAGE:3915647 5', mRNA sequence [BE622226]
2.01	DRD5	ref|Homo sapiens dopamine receptor D5 (DRD5), mRNA [NM_000798]
2.01	SLC4A1	ref|Homo sapiens solute carrier family 4 (anion exchanger), member 1 (Diego blood group) (SLC4A1), mRNA [NM_000342]
2.00	LOC100289580	ref|Homo sapiens uncharacterized LOC100289580 (LOC100289580), long non-coding RNA [NR_103774]
2.00	AK130532	gb|Homo sapiens cDNA FLJ27022 fis, clone SLV06258. [AK130532]

Supplementary Table S2. The representative genes that is lower expression in cytarabine-resistant HL60 cells than in parental HL60 cells.

Fold Change	Gene Name	Description
-780.41	XAGE1B	ref|Homo sapiens X antigen family, member 1B (XAGE1B), transcript variant a, mRNA [NM_001097594]
-31.16	CLC	ref|Homo sapiens Charcot-Leyden crystal galectin (CLC), mRNA [NM_001828]
-15.34	lnc-C5orf63-3	gb|DA821820 PERIC2 Homo sapiens cDNA clone PERIC2001885 5', mRNA sequence [DA821820]
-14.97	MITF	ref|Homo sapiens microphthalmia-associated transcription factor (MITF), transcript variant 1, mRNA [NM_198159]
-12.80	IKZF2	ref|Homo sapiens IKAROS family zinc finger 2 (Helios) (IKZF2), transcript variant 2, mRNA [NM_001079526]
-12.52	ENST00000433310	Unknown
-12.23	LINC01158	ref|Homo sapiens long intergenic non-protein coding RNA 1158 (LINC01158), long non-coding RNA [NR_037883]
-11.23	FGFR1	ref|Homo sapiens fibroblast growth factor receptor 1 (FGFR1), transcript variant 1, mRNA [NM_023110]
-10.11	LOC101928087	ref|PREDICTED: Homo sapiens uncharacterized LOC101928087 (LOC101928087), ncRNA [XR_250430]
-9.56	C22orf34	ens|chromosome 22 open reading frame 34 [Source:HGNC Symbol;Acc:HGNC:28010] [ENST00000400023]
-9.15	LOC143666	ref|Homo sapiens uncharacterized LOC143666 (LOC143666), long non-coding RNA [NR_026967]
-7.43	LY6K	ref|Homo sapiens lymphocyte antigen 6 complex, locus K (LY6K), transcript variant 1, mRNA [NM_017527]
-7.29	TCAM1P	ref|Homo sapiens testicular cell adhesion molecule 1, pseudogene (TCAM1P), non-coding RNA [NR_002947]
-6.86	IGLL1	ref|Homo sapiens immunoglobulin lambda-like polypeptide 1 (IGLL1), transcript variant 1, mRNA [NM_020070]
-5.94	CPXM1	ref|Homo sapiens carboxypeptidase X (M14 family), member 1 (CPXM1), transcript variant 1, mRNA [NM_019609]
-5.88	lnc-VAX1-1	linc|LNCipedia lincRNA (lnc-VAX1-1), lincRNA [lnc-VAX1-1:5]
-5.30	SLC4A11	ref|Homo sapiens solute carrier family 4, sodium borate transporter, member 11 (SLC4A11), transcript variant 2, mRNA [NM_032034]

-5.05	PAX8-AS1	ref|Homo sapiens PAX8 antisense RNA 1 (PAX8-AS1), transcript variant 2, long non-coding RNA [NR_047570]
-4.92	KCNH2	ref|Homo sapiens potassium voltage-gated channel, subfamily H (eag-related), member 2 (KCNH2), transcript variant 1, mRNA [NM_000238]
-4.79	LOC101927534	ref|PREDICTED: Homo sapiens uncharacterized LOC101927534 (LOC101927534), ncRNA [XR_245057]
-4.75	ENST00000390323	ens|immunoglobulin lambda constant 2 (Kern-Oz- marker) [Source:HGNC Symbol;Acc:HGNC:5856] [ENST00000390323]
-4.65	DLG3	ref|Homo sapiens discs, large homolog 3 (Drosophila) (DLG3), transcript variant 1, mRNA [NM_021120]
-4.56	ELANE	ref|Homo sapiens elastase, neutrophil expressed (ELANE), mRNA [NM_001972]
-4.42	PTPRS	ref|Homo sapiens protein tyrosine phosphatase, receptor type, S (PTPRS), transcript variant 1, mRNA [NM_002850]
-3.69	PLA2G3	ref|Homo sapiens phospholipase A2, group III (PLA2G3), mRNA [NM_015715]
-3.65	PRUNE2	ref|Homo sapiens prune homolog 2 (Drosophila) (PRUNE2), mRNA [NM_015225]
-3.55	AEBP1	ref|Homo sapiens AE binding protein 1 (AEBP1), mRNA [NM_001129]
-3.47	AGAP1	ref|Homo sapiens ArfGAP with GTPase domain, ankyrin repeat and PH domain 1 (AGAP1), transcript variant 1, mRNA [NM_001037131]
-3.46	MAGEA2B	ref|Homo sapiens melanoma antigen family A, 2B (MAGEA2B), mRNA [NM_153488]
-3.37	CARD10	ref|Homo sapiens caspase recruitment domain family, member 10 (CARD10), mRNA [NM_014550]
-3.21	GALC	ref|Homo sapiens galactosylceramidase (GALC), transcript variant 1, mRNA [NM_000153]
-3.12	SAP25	ref|Homo sapiens Sin3A-associated protein, 25kDa (SAP25), mRNA [NM_001168682]
-3.10	CADM3-AS1	ref|Homo sapiens CADM3 antisense RNA 1 (CADM3-AS1), long non-coding RNA [NR_037870]
-3.10	PDGFRL	ref|Homo sapiens platelet-derived growth factor receptor-like (PDGFRL), mRNA [NM_006207]
-3.03	PRG2	ref|Homo sapiens proteoglycan 2, bone marrow (natural killer cell activator, eosinophil granule major basic protein) (PRG2), transcript variant 1, mRNA [NM_002728]

-2.98	OBSL1	ref|Homo sapiens obscurin-like 1 (OBSL1), transcript variant 3, mRNA [NM_001173408]
-2.96	KIR3DL2	ref|Homo sapiens killer cell immunoglobulin-like receptor, three domains, long cytoplasmic tail, 2 (KIR3DL2), transcript variant 1, mRNA [NM_006737]
-2.96	ANKRD35	ref|Homo sapiens ankyrin repeat domain 35 (ANKRD35), transcript variant 1, mRNA [NM_144698]
-2.89	KIR2DS2	ref|Homo sapiens killer cell immunoglobulin-like receptor, two domains, short cytoplasmic tail, 2 (KIR2DS2), transcript variant 1, mRNA [NM_012312]
-2.86	FAM167B	ref|Homo sapiens family with sequence similarity 167, member B (FAM167B), mRNA [NM_032648]
-2.84	FGF9	ref|Homo sapiens fibroblast growth factor 9 (FGF9), mRNA [NM_002010]
-2.76	MEGF6	ref|Homo sapiens multiple EGF-like-domains 6 (MEGF6), mRNA [NM_001409]
-2.75	A_33_P3216621	Unknown
-2.68	GPSM1	ref|Homo sapiens G-protein signaling modulator 1 (GPSM1), transcript variant 1, mRNA [NM_001145638]
-2.64	CCDC140	ref|Homo sapiens coiled-coil domain containing 140 (CCDC140), mRNA [NM_153038]
-2.63	LOC102724601	ref|Homo sapiens uncharacterized LOC102724601 (LOC102724601), long non-coding RNA [NR_121197]
-2.62	lnc-AL590822.1-1	gb|UI-H-FE1-beg-g-15-0-UI.s1 NCI_CGAP_FE1 Homo sapiens cDNA clone UI-H-FE1-beg-g-15-0-UI 3', mRNA sequence [CA425772]
-2.58	PTPN14	ref|Homo sapiens protein tyrosine phosphatase, non-receptor type 14 (PTPN14), mRNA [NM_005401]
-2.57	BU963192	gb|AGENCOURT_10615922 NIH_MGC_141 Homo sapiens cDNA clone IMAGE:6744194 5', mRNA sequence [BU963192]
-2.55	ZNF337-AS1	ref|Homo sapiens ZNF337 antisense RNA 1 (ZNF337-AS1), transcript variant 2, long non-coding RNA [NR_126466]
-2.52	ENST00000455890	ens|long intergenic non-protein coding RNA 237 [Source:HGNC Symbol;Acc:HGNC:38166] [ENST00000455890]
-2.49	REEP1	ref|Homo sapiens receptor accessory protein 1 (REEP1), transcript variant 2, mRNA [NM_022912]

-2.48	KIR2DL2	ref|Homo sapiens killer cell immunoglobulin-like receptor, two domains, long cytoplasmic tail, 2 (KIR2DL2), mRNA [NM_014219]
-2.45	DLX4	ref|Homo sapiens distal-less homeobox 4 (DLX4), transcript variant 1, mRNA [NM_138281]
-2.44	DCAF4L1	ref|Homo sapiens DDB1 and CUL4 associated factor 4-like 1 (DCAF4L1), mRNA [NM_001029955]
-2.42	GP1BB	ref|Homo sapiens glycoprotein Ib (platelet), beta polypeptide (GP1BB), mRNA [NM_000407]
-2.41	AFAP1-AS1	ref|Homo sapiens AFAP1 antisense RNA 1 (AFAP1-AS1), antisense RNA [NR_026892]
-2.40	CCR7	ref|Homo sapiens chemokine (C-C motif) receptor 7 (CCR7), mRNA [NM_001838]
-2.39	OVOL2	ens|ovo-like zinc finger 2 [Source:HGNC Symbol;Acc:HGNC:15804] [ENST00000483661]
-2.39	PRSS57	ref|Homo sapiens protease, serine, 57 (PRSS57), mRNA [NM_214710]
-2.38	DGCR5	ref|Homo sapiens DiGeorge syndrome critical region gene 5 (non-protein coding) (DGCR5), transcript variant 1, non-coding RNA [NR_002733]
-2.38	HDAC9	ens|histone deacetylase 9 [Source:HGNC Symbol;Acc:HGNC:14065] [ENST00000406451]
-2.37	LYSMD4	ens|LysM, putative peptidoglycan-binding, domain containing 4 [Source:HGNC Symbol;Acc:HGNC:26571] [ENST00000496108]
-2.37	CCDC103	ens|family with sequence similarity 187, member A [Source:HGNC Symbol;Acc:HGNC:35153] [ENST00000331733]
-2.33	ZNF304	ref|Homo sapiens zinc finger protein 304 (ZNF304), transcript variant 2, mRNA [NM_020657]
-2.30	S100Z	ref|Homo sapiens S100 calcium binding protein Z (S100Z), mRNA [NM_130772]
-2.28	LOC100286922	ref|Homo sapiens DnaJ (Hsp40) homolog, subfamily B, member 3 pseudogene (LOC100286922), transcript variant 1, non-coding RNA [NR_037694]
-2.26	ENST00000413567	gb|K-EST0217412 L18POOL1n1 Homo sapiens cDNA clone L18POOL1n1-8-D04 5', mRNA sequence [CB158131]
-2.23	FBP1	ref|Homo sapiens fructose-1,6-bisphosphatase 1 (FBP1), transcript variant 1, mRNA [NM_000507]

-2.18	SMPDL3A	ref|Homo sapiens sphingomyelin phosphodiesterase, acid-like 3A (SMPDL3A), transcript variant 1, mRNA [NM_006714]
-2.17	lnc-ZC3H12B-3	linc|LNCipedia lincRNA (lnc-ZC3H12B-3), lincRNA [lnc-ZC3H12B-3:1]
-2.17	LOC647264	ens|long intergenic non-protein coding RNA 1198 [Source:HGNC Symbol;Acc:HGNC:49598] [ENST00000595532]
-2.15	lnc-BNIP1-1	linc|LNCipedia lincRNA (lnc-BNIP1-1), lincRNA [lnc-BNIP1-1:1]
-2.15	F13A1	ref|Homo sapiens coagulation factor XIII, A1 polypeptide (F13A1), mRNA [NM_000129]
-2.14	LXN	ref|Homo sapiens latexin (LXN), mRNA [NM_020169]
-2.11	LINC01422	ref|Homo sapiens long intergenic non-protein coding RNA 1422 (LINC01422), long non-coding RNA [NR_110540]
-2.09	WFDC5	ref|Homo sapiens WAP four-disulfide core domain 5 (WFDC5), mRNA [NM_145652]
-2.09	ENST00000532091	ens|heat shock 70kDa protein 8 [Source:HGNC Symbol;Acc:HGNC:5241] [ENST00000532091]
-2.09	COX8C	ref|Homo sapiens cytochrome c oxidase subunit VIIIC (COX8C), mRNA [NM_182971]
-2.09	ENST00000555032	Unknown
-2.08	C1orf226	ref|Homo sapiens chromosome 1 open reading frame 226 (C1orf226), transcript variant 2, mRNA [NM_001085375]
-2.06	LCK	ref|Homo sapiens LCK proto-oncogene, Src family tyrosine kinase (LCK), transcript variant 2, mRNA [NM_005356]
-2.06	PRSS8	ref|Homo sapiens protease, serine, 8 (PRSS8), mRNA [NM_002773]
-2.04	LOC101927056	ref|Homo sapiens uncharacterized LOC101927056 (LOC101927056), transcript variant 1, long non-coding RNA [NR_125395]
-2.02	CCDC28B	ref|Homo sapiens coiled-coil domain containing 28B (CCDC28B), transcript variant 2, mRNA [NM_024296]
-2.01	ENST00000566418	Unknown
-2.01	ATP8A2	ref|Homo sapiens ATPase, aminophospholipid transporter, class I, type 8A, member 2 (ATP8A2), mRNA [NM_016529]
-2.01	CASZ1	ref|Homo sapiens castor zinc finger 1 (CASZ1), transcript variant 1, mRNA [NM_001079843]
